# Scientific and Folk Theories of Viral Transmission: A Comparison of COVID-19 and the Common Cold

**DOI:** 10.3389/fpsyg.2022.929120

**Published:** 2022-06-28

**Authors:** Danielle Labotka, Susan A. Gelman

**Affiliations:** Department of Psychology, University of Michigan, Ann Arbor, MI, United States

**Keywords:** causal reasoning, illness understanding, COVID-19, folk theories, explanatory co-existence

## Abstract

Disease transmission is a fruitful domain in which to examine how scientific and folk theories interrelate, given laypeople’s access to multiple sources of information to explain events of personal significance. The current paper reports an in-depth survey of U.S. adults’ (*N* = 238) causal reasoning about two viral illnesses: a novel, deadly disease that has massively disrupted everyone’s lives (COVID-19), and a familiar, innocuous disease that has essentially no serious consequences (the common cold). Participants received a series of closed-ended and open-ended questions probing their reasoning about disease transmission, with a focus on causal mechanisms underlying disease contraction, transmission, treatment, and prevention; non-visible (internal) biological processes; and ontological frameworks regarding what kinds of entities viruses are. We also assessed participants’ attitudes, such as their trust in scientific experts and willingness to be vaccinated. Results indicated complexity in people’s reasoning, consistent with the co-existence of multiple explanatory frameworks. An understanding of viral transmission and viral replication existed alongside folk theories, placeholder beliefs, and lack of differentiation between viral and non-viral disease. For example, roughly 40% of participants who explained illness in terms of the transmission of viruses also endorsed a non-viral folk theory, such as exposure to cold weather or special foods as curative. Additionally, participants made use of competing modes of construal (biological, mechanical, and psychological) when explaining how viruses operate, such as framing the immune system response (biological) as cells trying to fight off the virus (psychological). Indeed, participants who displayed greater knowledge about viral transmission were significantly more likely to anthropomorphize bodily processes. Although comparisons of COVID-19 and the common cold revealed relatively few differences, the latter, more familiar disease elicited consistently lower levels of accuracy and greater reliance on folk theories. Moreover, for COVID-19 in particular, accuracy positively correlated with attitudes (trusting medical scientists and taking the disease more seriously), self-protective behaviors (such as social distancing and mask-wearing), and willingness to be vaccinated. For both diseases, self-assessed knowledge about the disease negatively predicted accuracy. The results are discussed in relation to challenges for formal models of explanatory reasoning.

## Introduction

Disease transmission is potentially a fruitful domain in which to examine how scientific and folk theories interrelate (Legare and Visala, 2011). People are highly motivated to learn about disease, as it has direct consequences for their own and loved ones’ well-being, yet the causal agents cannot be seen and the sources of information are highly varied—ranging from medical professionals and public health officials, to family, friends, and social media. Research on illness understanding indicates that germ theory, though historically a relatively recent discovery (Thagard, 1999), is so well entrenched in modern society that even preschool children understand germs as responsible for illness despite being unseen (Kalish, 1996; Blacker and LoBue, 2016). At the same time, however, non-germ theories of illness persist. An in-depth examination of illness theories in Hong Kong revealed that adults as well as children commonly endorsed folk beliefs about the causes of cold and flu, such as getting sick from being exposed to cold air, being drenched in rain or sweat, or eating too much deep-fried food (Au et al., 2008). Likewise, Mesoamerican cultures express the belief that an imbalance of hot and cold may lead to illness, based on a classification of objects, foods, illnesses, treatments, etc. into intrinsic properties of “hot” and “cold,” though these may not be related to temperature *per se* (García-Hernández et al., 2021). Moreover, belief that exposure to cold is a cause of illness has been documented in a range of cultural contexts (e.g., Helman, 1978; Sigelman et al., 1993; Baer et al., 1999, 2008; Toyama, 2019; Motta and Callaghan, 2020).

In the present work, we characterize scientific theories as reflecting contemporary science, and intuitive or folk theories as deviating from scientific consensus but not invoking supernatural causes (see Shtulman and Lombrozo, 2016, pp. 51, 52). Furthermore, we echo an important point noted by Motta and Callaghan (2020), that medically inaccurate beliefs may nonetheless have significant cultural, traditional, or historical value to those who hold them. Both folk and scientific theories spell out the ontology of a domain (i.e., the fundamental sorts of things there are in that domain) as well as the basic causal principles that are particular to that domain (see Carey, 1985; Wellman and Gelman, 1992; Au et al., 2008).

Motta and Callaghan (2020) found that endorsement of what they call “medical folk wisdom” (inaccurate beliefs about disease and other medically relevant topics, such as pregnancy) is pervasive in the U.S. Examples include believing that multivitamins boost immunity, that cold weather causes colds, that “starving” a fever can speed recovery, and that chicken soup and excess vitamin C cure colds. Furthermore, holding such beliefs is associated with placing less value on medical expertise, even controlling for a participant’s economic status, political orientation, and educational background—perhaps due to a knowledge deficit whereby people are unaware of the scientific evidence, or perhaps due to cognitive dissonance involving awareness of the competing medical stances (Motta and Callaghan, 2020).

Given that intuitive causal theories are common in the biological domain (Coley et al., 2017), an important question is how they relate to scientific theories. Folk theories can conflict with scientific theories— and which theories people endorse can have implications for their decision-making, health-promoting behaviors, and endorsements of public policy recommendations (Au et al., 2008; Shtulman and Harrington, 2016). Updating beliefs based on new scientific discoveries can affect people’s behaviors and policy endorsements regarding topics as wide-ranging as vaccinations to protect against disease, response to climate change, and means of preventing pregnancy (Hornsey et al., 2016; Motta and Callaghan, 2020). Moreover, understanding causal mechanisms may increase awareness of how behaviors can lead to positive health outcomes, which is vital to motivating a person to intend to take action (i.e., to move past Stage 1 of Prochaska and DiClemente’s transtheoretical model of behavior change [Prochaska, 2020]).

Classic models of cognitive development have assumed a “replacement model,” according to which old, incorrect theories are replaced as newer, more correct theories are introduced (e.g., Carey, 1985; Vosniadou, 1994). However, recent evidence suggests that scientific explanatory models do not necessarily replace prior intuitive theories, but rather, distinct causal models may co-exist within an individual’s understanding. For example, undergraduate students in Japan simultaneously report that illness is caused by germs *and* “resistance” (a folk-theoretic concept involving lessened vital power or force; Toyama, 2019), and adults in South Africa report that germs cause AIDS via unprotected sex *and* that not respecting one’s neighbor may lead to illness via witchcraft (Legare and Gelman, 2008). This “coexistence model” suggests that people’s knowledge in a domain may not be as coherent as has often been assumed, and that instead how people reason may reflect the context in which a problem is framed (Raman and Gelman, 2005; see also Giménez and Harris, 2005; Astuti and Harris, 2008; Rosengren et al., 2014). To the extent that intuitive theories persist, this also suggests that instigating conceptual change may be a challenging proposition, as simply introducing a new, more explanatory theory is unlikely to be sufficient. This is particularly the case if motivations for holding onto folk theories include factors such as values, ideologies, and political orientations (e.g., Hornsey et al., 2016).

More generally, co-existence models have been found for a range of scientific concepts across multiple domains, including astronomy, evolution, mechanics, thermodynamics, etc. (Kelemen and Rosset, 2009; Shtulman and Valcarcel, 2012; Shtulman and Harrington, 2016). Legare et al. (2012) note that there are various ways that these seemingly competing beliefs may relate to one another. They may reflect: (a) target-dependent thinking, whereby different accounts are used to explain different aspects of a phenomenon (e.g., “Witchcraft can cause a disease that looks like AIDS”); (b) synthetic thinking, whereby two different explanations are combined but without any explicit integration (e.g., “It might be witchcraft and having unprotected sex”); or (c) integrative thinking, whereby the two explanatory frameworks are combined into a single explanation (e.g., “A witch can put you in the way of viruses and germs”) (all examples here are from Legare and Gelman, 2008).

Although explanatory co-existence has been observed in multiple domains, we still know little about when, why, and how such beliefs may interrelate. As Shtulman and Legare (2020) note, “The persistence of intuitive theories seems to be inevitable, but the dynamics of when and how they are deployed remains a fruitful topic for further investigation.” These questions are the focus of the present investigation.

### The Context of COVID-19

COVID-19 is an especially opportune context for examining explanatory co-existence, given its novelty, danger, and variability. Because it is a *new* disease about which much was unknown when it first emerged (and much is still unknown, at the time of this writing), there is less of an established scientific knowledge base, and correspondingly a greater variety of competing accounts from which reasoners can select. Additionally, as knowledge accrued, expert advice shifted (e.g., upon learning that the virus is airborne and fomites an unlikely source of transmission, masks became important and placing mail in “quarantine” less advised). Because it is a remarkably *dangerous* disease that emerged rapidly and affected nearly everyone in some capacity, it may evoke strong emotional responses and heighten people’s reliance on emotions and attitudes. The COVID-19 pandemic massively disrupted daily life, and during its initial emergence, much was still unknown even in the scientific and medical communities (Lipsitch et al., 2020). In such contexts, rational consideration of evidence may be lowered, given the potential role of motivated cognition when reasoning in the face of high threat (Loewenstein and Lerner, 2003; Gelman, 2011).

Related to both novelty and danger, COVID-19 has presented new, unusual, and potentially alarming physical and psychological symptoms, including loss of smell, cognitive impairment, a mismatch between a person’s subjective symptoms and their objective bodily state (feeling fine while being close to collapse due to oxygen insufficiency; so-called “happy hypoxemia”), and symptoms that may persist for weeks or months (“long COVID” and post-COVID-19 syndrome) (Aiyegbusi et al., 2021). Finally, COVID-19 is also a remarkably *variable* disease (Weill Cornell Medicine, 2020): variable in symptoms (ranging from asymptomatic to death), degree of risk (including unpredictable “super-spreader” events), and new variants, associated with different risks of transmission, immune invasion, and/or incubation periods (Del Rio et al., 2022). This variability may elicit widely varying responses as a function of one’s own perceived risk.

Altogether, this portrait of COVID-19 highlights that when the pandemic emerged: (a) a great deal was unknown, (b) the perception of danger could be high, but the risk to any individual was also potentially quite variable, and (c) information available to the public was characterized by rapidly shifting expert knowledge, opinion, and advice. It may be that such ingredients provided an opportunity for more varied and even polarized responses, including degree of trust in experts. Deference to experts is a critical component of reasoning about complex causal systems (Keil, 2006), and factors that influence how people assess their trust in that expertise is likely to play a role.

At the same time, COVID-19 presents a case where scientific and folk explanations may converge (albeit not completely). Shtulman (n.d.) argues that several phenomena that have been prominent during the COVID-19 pandemic (mask-wearing, social distancing, sanitation, diagnostic testing, treatment, and vaccinations) are in broad strokes consistent with both scientific and folk explanations, and that this convergence may help to explain why folk explanations persist in the face of scientific information. For example, although non-experts often misunderstand how vaccines work (e.g., they often report an “anti-virus” model of vaccines, whereby vaccines are thought to attack the virus directly, rather than to stimulate the immune system; Jee et al., 2015), many novices as well as experts believe that vaccines are effective in reducing disease. Accordingly, much messaging with regard to COVID-19 could simultaneously support folk theories as well as scientific theories.

Since March of 2020, there has been an outpouring of psychological research on COVID-19. Although much of this work is not directly relevant to causal theories (e.g., focusing instead on behavioral consequences of quarantining, remote schooling, and mask-wearing), studies that have examined beliefs about viral transmission suggest that adults in the U.S. hold a mixture of accurate and inaccurate beliefs, and that their beliefs relate to perceptions of risk, health-related decision-making, and interest in being vaccinated (see DeJesus et al., 2021; Leotti et al., 2021, for research on children’s beliefs). For example, in a sample of U.S. residents tested in October/November of 2020, Murray et al. (2021) assessed people’s knowledge about germs, asking them to judge as true or false a range of statements either consistent with scientific theory (e.g., “Being depressed can make a person sick”; “Germs enter the body through the eyes”) or inconsistent with scientific theory but consistent with naïve theory (e.g., “Water kills germs”; “Vitamins and minerals can cure viruses”). They found that individuals who had more knowledge about viral disease were more likely to make decisions consistent with guidelines from the Centers for Disease Control and Prevention (CDC) and expressed less cognitive conflict. They also found that endorsement of preventive behaviors correlated with wanting a vaccine. Moreover, in a two-wave study conducted in March and December of 2020, political conservatism was linked to more misconceptions regarding COVID-19 (e.g., “The seasonal flu is just as dangerous as the coronavirus.”), as well as lower perceptions of risk, lower likelihood of engaging in mitigation behaviors, and greater vaccine hesitancy (Pennycook et al., 2021). This is consistent with other data showing that political conservatism relates to vaccine hesitancy (El-Mohandes et al., 2021). At the same time, positive or negative feelings toward scientists have been found to play a greater role in COVID-19 attitudes and protective behaviors than political partisanship (Sanchez and Dunning, 2021).

### The Present Study

The present study was designed to explore the co-existence of biomedical and folk theories of COVID-19 (and the common cold, as comparison), with a focus on how people understand and explain unobservable viral transmission processes. Within this broader goal, there were four specific research questions (described in more detail below): (1) How do people understand aspects of viral transmission that are non-obvious or “below the surface”? (2) What is the ontological status of viruses? (3) How coherent are people’s beliefs about viral transmission, and do we see evidence of multiple explanatory frameworks? (4) How do beliefs about COVID-19 compare to beliefs about the common cold? We probed these understandings, and the language used to express them, by means of a rich set of open-ended questions and “why/how” questions in order to elicit participants’ in-depth reasoning and explanations. This provides complementary evidence to methods such as reaction times (Shtulman and Valcarcel, 2012), mouse-tracking (Murray et al., 2021), or neuroimaging (Masson et al., 2014), which focus on rapid, real-time processing of multiple explanatory accounts.

#### Non-obvious Aspects of Viral Transmission

We were particularly interested in hidden or counter-intuitive aspects of viral transmission that are diagnostic of the underlying biological processes. Although illness itself may have observable signs (a rash, a runny nose), and illness transmission may have observable signs (spray from a sick person’s sneeze landing on another person), neither illness nor transmission need be observable, and the biological processes responsible for transmission and illness are themselves not available to direct visual inspection. In order to make accurate predictions of risk, one needs to understand a number of non-obvious features of disease, such as that carriers may be asymptomatic, that seemingly innocuous activities can transmit disease, and that viruses reproduce within the body over time.

#### Ontological Status of Viruses

We were also interested in assessing the ontological status of viruses, as ontological frameworks can have direct and indirect consequences for inferences and decision-making. For example, whether children think about germs in terms of biological vs. mechanical processes has direct consequences for their ability to make appropriate inferences and decisions about how to prevent disease spread (Au et al., 2008; Weisman and Markman, 2017). Viruses have some but not all features associated with independent living entities, and thus there is no consensus among scientists as to whether viruses are alive (Villarreal, 2004). Nonetheless, one can ask which properties people attribute to viruses. Key to an accurate understanding is recognizing that viruses can replicate within the cell of a living host and can be destroyed by soap and antimicrobial pesticides (Au et al., 2008). In this respect, viruses engage in biological processes (i.e., they are not inert entities that simply engage in mechanical processes; this is an understanding that does not appear to be available to young children; Solomon and Cassimatis, 1999). At the same time, viruses differ from other living organisms, because they are parasitic on the host’s body, and when outside the host’s body they cannot function and ultimately will not remain viable. Furthermore, unlike animals, viruses do not have psychological properties such as intentions or goals.

#### Coherence and Coexistence

To examine coexistence, we tracked misconceptions, including non-viral folk theories (e.g., that cold weather causes illness), and how often they overlapped with accurate scientific knowledge. We also characterized the implicit frameworks or modes of construal that participants used in their explanations, including biological, mechanical, and intentional forms of reasoning. For the latter, we examined the extent to which participants engaged in anthropomorphic framing of viral processes (e.g., talking of the COVID-19 virus searching for people to infect, or talking of the immune system fighting the virus), which may lead to insights as well as distortions, and affect an individual’s emotional engagement (Martin, 1991; Keil, 2006; Osbeck and Nersessian, 2011, 2013). Of particular interest was how often participants made use of multiple such frameworks.

#### COVID-19 Compared to the Common Cold

Participants were questioned about either COVID-19 or the common cold, as a comparison illness. The biological mechanisms for these different viral illnesses are in broad strokes quite similar, as both are viral diseases with similar transmission processes. However, the two diseases differ dramatically in familiarity (at the time of test, COVID-19 was in its first year, and had been labeled a pandemic only a few months prior, whereas the common cold would have been familiar throughout participants’ lives), consequences (COVID-19 potentially results in serious disease or death, whereas the common cold causes only mild symptoms that resolve within a few days), and politicization (surveys consistently show that for those in the U.S., political beliefs predict COVID-19 behaviors, such as social distancing, and likelihood of being vaccinated, Grossman et al., 2020; Hamel et al., 2020, whereas there are to our knowledge no comparable differences in how seriously people consider the common cold to be).

#### Additional Questions

In addition to assessing knowledge of viruses and viral transmission, we included a series of questions to assess participant’ attitudes toward COVID-19, their self-reported protective behaviors, and various experiential and demographic variables (e.g., whether they personally knew someone who had been diagnosed with COVID-19).

The research questions, coding, analyses, and participant exclusion criteria were pre-registered in AsPredicted: “Adults’ Biological Beliefs Concerning COVID-19 Disease Transmission” (#43174) and “Coding and Analysis Amendment to AsPredicted #43174” (#68931). It should be noted that due to a typographical error in the pre-registration, it stated residing in the U.S. as an exclusion, whereas we intended to indicate that *not* residing in the U.S. would be an exclusion.

## Materials and Methods

### Participants

The final sample included 238 adults recruited via MTurk and randomly assigned to condition (*n* = 119 COVID-19 condition, *n* = 119 Cold condition). Participant ages ranged from 20 to 69 years (*M* age 38.12; 111 women, 125 men, and 2 did not report gender). We had preregistered gathering a final sample minimum size of *n* = 120 per condition, but due to an error in identifying exclusions (i.e., a small number of exclusions due to duplicate IP addresses were not identified until well after the data were collected), the full sample fell slightly below the desired sample size.

This final sample included 115 parents and 123 non-parents. Self-reported race/ethnicity was white (*n* = 190), Black or African-American (*n* = 20), Hispanic or Latino/a (*n* = 11), Asian or Asian American (*n* = 10), Native American (*n* = 4), South Asian (*n* = 2), Middle Eastern (*n* = 1), multi-racial (*n* = 2), and unreported (*n* = 1). (Numbers exceed 238 because participants could select more than one category.) Education levels ranged from high school or equivalent through to professional degree, with median level of education being an Associate’s (two-year) degree. Annual income ranged from less than $15,000 to over $85,000 (the highest option provided), with median level $45,000–$65,000. Participants’ self-reported zip codes indicated they resided in 41 different states (3 not reported). We did not have individual participants’ voting behavior; however, voting behavior in their zip code in the 2020 U.S. presidential election (see section “Materials” section below), calculated as a Biden-Trump difference score, ranged from –100% to +92%, with a mean of +10.3%. There were no significant differences between participants in the COVID-19 versus Cold conditions in age, gender, education, income, voting behavior of their zip code in the 2020 election, or having children, all *p*s > 0.17.

Forty-five additional MTurk workers were tested but were excluded from the final sample due to duplicate IP addresses (*n* = 5), apparent low English comprehension (*n* = 1), and identification as bots (*n* = 39; e.g., by providing nonsensical responses to open-ended questions, the same response to all open-ended questions [e.g., “GOOD”], or responses that had been cut-and-pasted from the internet). All participants (final sample and exclusions) completed their surveys in June and July of 2020.

### Materials

The survey included questions assessing knowledge and beliefs regarding viral transmission, knowledge self-appraisal, COVID-19 attitudes (COVID-19 survey only), protective behaviors (COVID-19 survey only), conversations with their children about illness (parents only), and demographics. Each of these question sets is described below.

#### Viral Transmission

The primary test instrument was an in-depth survey assessing various aspects of knowledge and beliefs about COVID-19 or the common cold (see Table 1). Participants were first asked several open-ended questions to assess their general knowledge (e.g., how the disease is contracted, typical symptoms). Then they received a series of several dozen close-ended and open-ended questions designed to assess understanding of the causal mechanisms taking place when a virus is spread from one person to another. Questions focused on unobservable processes, including asymptomatic carriers, transmission from seemingly innocuous activities (e.g., singing, picking up a package), delays in symptom onset, asymptomatic hosts, and increases in viral load over time. The instrument also included questions designed to assess folk theories of viral disease (e.g., that exposure to cold weather can cause illness, or that special foods or vitamins can be curative). Several of the questions were adapted from prior research: The “grow” and “need food” items were adapted from Solomon and Cassimatis (1999); the “dead sick” and “how kill” questions were adapted from Au et al. (2008); the “symptom delay” question was adapted from Raman and Gelman (2007); the viral replication questions (“time lag,” “sick all over,” “more germs,” and “inside-outside”) were adapted from Au and Romo (1996, 1999).

**TABLE 1:** Viral transmission survey (COVID-19 version), with concepts and items in order of presentation.

Concept	Item	Wording	Coding
General knowledge	Effects	Please briefly explain how COVID-19 has affected your life.	#
	Symptoms	What are the symptoms of COVID-19?	#
	Contract	How do people contract COVID-19?	OE
	Protect	What can you do to protect yourself and other people from getting COVID-19?	OE
	Get better	If someone contracted COVID-19, how would they get better?	OE
Introduction to virus	(N/A)	This is a picture of the virus that causes COVID-19. Some of our questions will refer to this virus. Sometimes we refer to this as “the COVID-19 virus,” or “COVID-19 germs,” for short.	
Invisibility	Size	In real life, how big is a COVID-19 virus? [Too tiny to see with just your eyes, the size of a speck of dust, the size of a pea, the size of an orange]	AC: Too tiny to see
Biological features	Grow	Imagine a single COVID-19 virus. Can a COVID-19 virus grow bigger?	AC: No
	Move by itself	Can a COVID-19 virus move by itself?	AC: No
	Need food	Does a COVID-19 virus need food?	AC: No
Alive/dead	Can die	Can a COVID-19 virus die?	IN
	Alive	Please indicate whether each of the following is alive or not alive. [6 items including: an animal, a plant, a non-living natural kind, a moving artifact, a simple artifact, and “a COVID-19 virus”]	IN
		You said that a COVID-19 virus is [is not] alive. Why? [Why not?]	OE
	Dead sick	If a COVID-19 virus is dead, can it still make people sick?	AC: No
	How kill	How can you kill a COVID-19 virus?	OE
	Kill shoes	Can you kill COVID-19 germs by stepping on them with your shoes?	AC: No
	Kill freezer	Can you kill COVID-19 germs by putting them in the freezer?	AC: No
	Wash out	If COVID-19 germs get in your mouth, can you wash them out by drinking a big glass of water?	AC: No
	Dangerous alive or dead	Is a COVID-19 virus dangerous only when it’s alive, or is it dangerous even after it’s dead?	IN
Delayed onset	Symptom delay	Imagine a woman who was coughed on by someone who had COVID-19. How long would it take before she would start to feel sick? (Right away, later that same day, the next day, a few days later, one to two weeks later)	AC: A few days later or 1-2 weeks later
Viral replication	Time lag	Some COVID-19 germs got inside a man’s body. He felt okay for a few days. But then later he started to feel sick, all over his whole body. His head ached and his throat hurt and he had trouble breathing – all at the same time. Why did it take a few days for him to feel sick after the COVID-19 germs got inside his body?	OE
	Sick all over	How did the COVID-19 germs make him feel sick in so many parts of his body at the same time?	OE
	More germs	One day, a man was feeling very sick, so he went to the hospital. He stayed in a room that was very, very clean. Over the next few days, there were more and more COVID-19 germs in his body. How did that happen? Why were there more COVID-19 germs in his body?	OE
	Inside-outside	Did the additional COVID-19 germs come from inside his body or from outside his body?	AC: Inside
Fomites	Package	Suppose someone who was sick with COVID-19 coughed on a package, and their germs got all over the package. Do you think someone else could get COVID-19 by picking up the package?	AC: Yes
		Why or why not?	OE
	Package delay	What if the package stayed on a shelf for a whole week, and then someone picked it up – could they get COVID-19 by picking up the package?	AC: No
		Why or why not?	OE
Asymptomatic disease	Asymptomatic	Imagine a woman who feels great. She’s not coughing or sneezing. She doesn’t have a fever or headache. Could she have COVID-19?	AC: Yes
		Why or why not?	OE
	Asymptomatic transmit	Could she give someone else COVID-19?	AC: Yes
		How or why not?	OE
Points of entry	Foot	What if someone got COVID-19 germs on the bottom of their foot but not inside their body. Could that make them sick?	AC: No
	Nose	What if someone got COVID-19 germs in their nose. Could that make them sick?	AC: Yes
	Eyes	What if someone got COVID-19 germs in their eyes. Could that make them sick?	AC: Yes
Transmission risks	(N/A)	Consider a person who has COVID-19. For each of these activities, what is the likelihood that it would transmit COVID-19 to someone else? (could not transmit, very unlikely to transmit, neither likely nor unlikely to transmit, very likely to transmit)	
	Sneeze	Sneezing	AC: Yes
	Cough	Coughing	AC: Yes
	Candles	Blowing out birthday candles	AC: Yes
	High-five	Giving someone a high-five	AC: Yes
	Sing	Singing together	AC: Yes
	Cards	Playing cards	AC: Yes
	Phone	Talking to someone on the phone	AC: No
	Door	Standing on opposite sides of a glass door	AC: No
	Park	Sitting in different areas in a big park	AC: No
Reinfection	Get again	If someone gets COVID-19 once and then gets better, can they get it again or not?	AC: Yes
		Why or why not?	#
Folk beliefs	Foods prevent	Are there any foods that can stop you from getting COVID-19?	AC: No
		Why or why not?	OE
	Summer	Does COVID-19 go away in the summertime, when the weather gets hot?	AC: No
		Why or why not?	OE
Vaccines	Vaccine Knowledge	As you may know, scientists are working on developing a vaccine for COVID-19. How do vaccines work? How would a COVID-19 vaccine protect people?	OE
	Vaccine want	If a COVID-19 vaccine is developed, would you like to get the vaccine? [7-point scale]	IN
Causal mechanisms	(N/A)	Here are some behaviors that are good ways to protect yourself from COVID-19. For each one, please briefly explain why you think it helps.	
	Proximity	Don’t stand too close to someone who is sick.	OE
	Shake hands	Don’t shake hands with someone who is sick.	OE
	Mask	Wear a mask.	OE
	Wash hands	Wash your hands.	OE
	Wash hands with water	If you washed your hands with water but not soap, would that protect you from COVID-19? If so, how? If not, why not?	OE
	Touch face	Don’t touch your face.	OE
	Clean surface	Clean the countertop.	OE

*The Coding column indicates whether responses were close-ended items included in the Accuracy composite (AC; with correct response indicated), were close-ended items analyzed individually (IN), were open-ended questions coded for content (OE), or were not coded (#).*

#### Knowledge Self-Appraisal

Twice during the viral transmission survey, once at the beginning and again at the end, participants were asked, “On a scale from 1 to 5, please estimate how knowledgeable you are about how the COVID-19 virus works” [COVID-19 condition], or “On a scale from 1 to 5, please estimate how knowledgeable you are about how cold viruses work” [Cold condition]. Responses could range from 1 (Not at all knowledgeable) to 5 (Extremely knowledgeable).

#### COVID-19 Attitudes

Participants in the COVID-19 condition received three questions regarding their attitudes toward COVID-19, adapted from the Pew Research Center (n.d., 2020a,2020b): “How much do you think social distancing measures helped to slow the spread of coronavirus in the U.S.?” (4-point scale from Helped a lot to Have not helped at all); “How much of a threat, if any, is the coronavirus outbreak for the health of the U.S. population as a whole?” (3-point scale from A major threat to Not a threat); and “How much confidence, if any, do you have in medical scientists to act in the best interests of the public?” (4-point scale from A great deal of confidence to No confidence at all). All scales were coded so that higher scores indicated more serious attitudes (i.e., social distancing helped a lot; the coronavirus outbreak is a major threat; great deal of confidence in medical scientists).

#### Protective Behaviors

Participants in the COVID-19 condition were asked how often they engaged in each of the following behaviors intended to prevent COVID-19 transmission, on a 3-point scale (have done, have considered doing, have not considered doing) (those marked with an asterisk (*) were adapted from GALLUP, n.d., 2022): *Avoided going to public places, such as stores or restaurants; *avoided small gatherings of people, such as with family or friends; *avoided traveling by airplane, bus, subway, or train; *stocked up on food, medical supplies, or cleaning supplies; worn a mask on your face when outside your home; maintained 6 feet of distance from people outside your household; practiced frequent handwashing; avoided touching your eyes, nose, and mouth with unwashed hands; avoided contact with people who are sick (even inside your home); covered coughs and sneezes; cleaned and disinfected frequently touched surfaces; cleaned off/wiped down groceries; *worked from home; *stayed home from work and unable to work. All items were scored such that higher numbers indicate higher protection.

#### Parent Illness Conversations

Participants who indicated that they had children or stepchildren were asked three questions regarding the frequency and nature of their discussions of illness with their children (either COVID-19 or cold, depending on the condition), on a 5-point scale from Never to Almost all the time. Specifically, they were asked how often they discussed each of the following topics with their children: “What is COVID-19 [a cold]?”, “What can you do so that you and others don’t get COVID-19 [colds]?”, and “What happens if you get sick with COVID-19 [a cold]?” We refer to these as definition, prevention, and consequences, respectively.

#### Demographics

These questions assessed age, highest education level, profession, family’s combined yearly income, children or stepchildren, marital status, gender, race/ethnicity, and zip code. Zip code was used to determine voting behavior in the 2020 U.S. presidential selection in that community. Voting behavior for that zip code was available for 92% of participants; for the remainder, county-level voting behavior was determined. Participants were also asked: “Do you personally know someone who has been diagnosed as having COVID-19 by a health care provider?”

### Procedure

Participants completed a Qualtrics survey regarding either COVID-19 or the common cold (randomly determined). Participants were instructed not to consult other sources when filling out the survey. The survey was restricted to those residing in the U.S. Participants were paid $3 each.

### Coding

#### Composite Scales

We pre-registered two composite scales, assessing accuracy and self-appraised knowledge. The *Accuracy* scale was an average of responses to 29 closed-ended items (yes/no or multiple choice), each of which was coded as correct or incorrect (see Table 1). The *Self-knowledge* scale was an average of the 2 knowledge self-appraisal items.

In addition, we created three composite scales that were not pre-registered but were theoretically motivated and had acceptable alphas (reported below), regarding: COVID-19 attitudes, protective behaviors, and (for parents only) conversations with their children about illness. The *COVID-19 attitudes* scale averaged the three items after converting the “threat” question to a 4-point scale to match the other two items, and reverse-coded so that higher scores indicated a more serious attitude (higher likelihood of thinking that social distancing helped slow the spread of coronavirus, viewing the coronavirus outbreak as more of a threat, and having greater confidence that medical scientists act in the best interests of the public). The *Protective behaviors* scale was an average of the 14 protective behaviors, reverse-coded so that higher scores indicated higher likelihood of engaging in the behavior. Finally, the *Parent illness conversation* scale averaged the three items, with higher scores indicating higher frequency of conversation.

Cronbach’s alphas were all at acceptable levels: Accuracy (COVID-19 α = 0.85, Cold α = 0.79); Self-knowledge (COVID-19 α = 0.91, Cold α = 0.86); COVID-19 attitudes (COVID-19 condition only, α = 0.80); Protective behaviors (COVID-19 condition only, α = 0.90); Parent illness conversations (COVID-19 α = 0.92, Cold α = 0.70).

#### Qualitative Coding

As pre-registered, open-ended responses were coded to assess 12 distinct concepts (see Table 2). For each coding category, 20% of responses were coded by two independent coders, and disagreements were resolved by discussion. Here we provide percent agreements and Krippendorff’s alphas for each. The first six codes were drawn from Au et al. (2008): (a) Germ survival or death (germs can live or die) (99% agreement, α = 0.89); (b) Germ replication (germs increase in number, not in size) (98% agreement, α = 0.95); (c) Explicit germ movement (germs come from sources such as saliva or breath; germs move from one person to another, or from outside to inside the body) (90% agreement, α = 0.79); (d) Implicit germ movement (alluding to mechanisms such as sneezing or contact that transfer contaminants without mentioning germs explicitly) (87% agreement, α = 0.70); (e) Folk beliefs (e.g., people get sick by exposure to cold air) (98% agreement, α = 0.85); and (f) Points of entry (viruses can enter the body via the nose, mouth, or eyes) (98% agreement, α = 0.85). Four codes captured additional beliefs or understandings of theoretical interest: (g) Viruses require a host, or function differently inside vs. outside the body (97% agreement, α = 0.86); (h) Immune system response (including mention of antibodies) (98% agreement, α = 0.91); (i) Vaccines as preventive (97% agreement, α = 0.91); (j) Vaccines as curative (100% agreement, α = 1.00). The final two codes captured (k) Anthropomorphism (referring to viruses or viral processes [e.g., immune system response] as if they have agentive, animate, biological, or psychological properties; these could include genuine beliefs, metaphoric language, analogy, or pretense) (95% agreement, α = 0.67); and (l) Undifferentiated illness (referring to a virus as bacteria; referring to COVID-19 as the flu) (99% agreement, α = 0.64). Regarding the Anthropomorphism code, disagreement largely concerned words that were in a “gray area” between anthropomorphism and literal language use (e.g., sit, move, travel, build, boost, allow, protect). Following discussion of disagreements, the coders determined to use a more conservative metric and exclude such terms from the coding category. Nonetheless, these lower alphas should be kept in mind as limitations in use and interpretation of these measures.

**TABLE 2:** Qualitative coding, coding categories and examples.

Coding category	Definition	Example
Germ survival or death	Germs can live or die.	Hot water and rubbing would probably kill most of the cold virus but not as much as soap.
Germ replication	Germs increase in number (not in size).	The germs had to multiply over the course of the few days before they reached a significant quantity in order to cause bodily harm and therefore cause symptoms.
Explicit germ movement	Germs come from sources such as saliva or breath, or germs move from one person to another, or from outside to inside the body.	Prevents the other person from transmitting the virus through respiratory particles.
Implicit germ movement	Mechanisms such as sneezing or contact that transfer contaminants, without mentioning germs explicitly.	You are less likely to get the respiratory droplets spread to you.
Folk beliefs	Non-viral mechanisms, such as getting sick by exposure to cold air.	It has been proven that Vitamin C is great for your immune system that can keep you from contracting colds. Some foods rich in Vitamin C are citrus.
Points of entry	Virus can enter the body via the nose, mouth, or eyes.	Wearing a mask helps to block droplets from getting into your nose and mouth.
Viruses require a host	Virus requires a host, or functions differently inside vs. outside the body.	The germs could not live that long outside of a host body.
Immune system response	Mention of immune system or antibodies.	Your antibodies can kill it.
Vaccines as preventive	Vaccines can prevent disease.	It would protect people by taking a virus that is weakened or dead and putting it inside the body so the body can recognize it so that if they do get the virus, the body will know what to do to protect the person instead of going into overdrive.
Vaccines as curative	Vaccines can cure disease.	Vaccines are basically medicine with the actual virus (dead) and some other things in it.
Anthropomorphism	Referring to viruses or viral processes as if they have agentive, animate, biological, or psychological properties	When they [viruses] get in your system they try to bond with other cells and the body takes it as an attack.
Undifferentiated illness	Equating virus and bacteria, or different illnesses (e.g., COVID-19 and flu)	It [cold virus] is a bacteria.

## Results

The results are organized into four main sections. The first three sections correspond to the first three specific research questions listed in the Introduction (see “The present study”), regarding: non-obvious aspects of viral transmission; ontological status of viruses; and coherence and co-existence. The fourth specific research question (regarding COVID-19 compared to the common cold) is integrated into each of those three sections. Finally, the fourth section examines how attitudes, self-reported behaviors, and demographics link to beliefs and explanations.

### Non-obvious Aspects of Viral Transmission

To address the question of how people understand aspects of viral transmission that are non-obvious or “below the surface,” we focused on participants’ performance on the Accuracy Composite. Scores were significantly above chance (0.50) in both conditions, *t*s > 20, *p*s < 0.001, but higher in the COVID-19 condition, *M* (*SD*) = 0.84 (0.16), than the Cold condition, *M* (*SD*) = 0.79 (0.15), *t*(236) = 2.33, *p* = 0.021. Table 3 provides the data for each item. Comparisons against chance were conducted via binomial tests, with alphas set to <0.01 due to the multiple tests. Of the 29 items, all 29 exceeded chance in the COVID-19 condition, and 26 exceeded chance in the Cold condition. Condition differences were obtained on 4 of the 29 items, by Chi-square tests (with alphas again set to <0.01 due to the multiple tests): foods prevent, virus needs food, summer, and symptom delay, all of which were significantly higher when asked about COVID-19 than cold. These results suggest that greater experience with an illness does not necessarily lessen reliance on folk theories.

**TABLE 3:** Accuracy composite, proportion correct per concept and item, as a function of condition (COVID-19 vs. cold).

Concept	COVID-19	Cold	Item (see Table 1 for wording)	Response coded as correct	COVID-19	Cold	COVID-19 vs. Cold
Invisibility	0.87	0.83	Size	Too tiny to see with just your eyes	0.87**	0.83**	n.s.
Biological features	0.74	0.65	Grow	No	0.73**	0.69**	n.s.
			Move by itself	No	0.66**	0.56	n.s.
			Need food	No	0.83**	0.68**	.01
Alive/dead	0.88	0.82	Dead sick	No	0.79**	0.76**	n.s.
			Kill shoes	No	0.96**	0.91**	n.s.
			Kill freezer	No	0.84**	0.73**	n.s.
			Wash out	No	0.91**	0.89**	n.s.
Delayed onset	0.72	0.47	Symptom delay	A few days later or 1–2 weeks later	0.72**	0.47	<0.001
Viral replication	0.87	0.96	Inside-outside	Inside	0.87**	0.96**	n.s.
Fomites	0.83	0.87	Package	Yes	0.87**	0.92**	n.s.
			Package delay	No	0.78**	0.81**	n.s.
Asymptomatic disease	0.82	0.70	Asymptomatic	Yes	0.82**	0.70**	n.s.
			Asymptomatic transmit	Yes	0.82**	0.69**	n.s.
Points of entry	0.88	0.86	Foot	No	0.75**	0.80**	n.s.
			Nose	Yes	0.96**	0.94**	n.s.
			Eyes	Yes	0.92**	0.85**	n.s.
Transmission risks	0.87	0.85	Sneeze	Yes	0.92**	0.96**	n.s.
			Cough	Yes	0.96**	0.91**	n.s.
			Candles	Yes	0.88**	0.87**	n.s.
			High-five	Yes	0.89**	0.90**	n.s.
			Sing	Yes	0.89**	0.90**	n.s.
			Cards	Yes	0.86**	0.82**	n.s.
			Phone	No	0.88**	0.80**	n.s.
			Door	No	0.81**	0.80**	n.s.
			Park	No	0.72**	0.76**	n.s.
Reinfection	0.66	0.80	Get again	Yes	0.66**	0.80**	n.s.
Folk beliefs	0.87	0.64	Foods prevent	No	0.90**	0.61	<0.001
			Summer	No	0.83**	0.66**	0.003

**Significantly different from chance by binomial test, p ≤ 0.001. COVID-19 vs. Cold comparisons were conducted using chi-square tests. For comparisons against chance and across conditions, ps > 0.01 were not reported, due to the multiple tests.

Despite this overall excellent performance, some items generated more errors. If we identify concepts for which at least one-quarter of the respondents provided an incorrect answer (an arbitrary cut-off, but representing a notable subset of the sample), we see that these include the following misconceptions (for both illnesses unless otherwise noted): that a virus can grow bigger and move by itself, that a virus needs food (Cold only), that putting a virus in a freezer can kill it (Cold only), that symptoms will appear within a day, that someone without symptoms cannot be sick or transmit illness (Cold only), that a virus on the bottom of the foot can make one sick (COVID-19 only), that one can get sick by sitting in different areas of a big park than another person (COVID-19 only), that one cannot get the illness more than once (COVID-19 only), that there are foods that can prevent illness (Cold only), and that illness goes away in the summertime when it gets hot (Cold only). These items suggest that a sizable subset of people are uncertain about how the (invisible) viral transmission process works. Each of these concepts is examined in more depth in the coding of the open-ended responses.

### Ontological Status of Viruses

To examine the ontological status of viruses, and the extent to which they are understood as quasi-living agents, we focused on three additional questions regarding the life status of COVID-19 or cold viruses: Are they alive? Can they die? And are they dangerous only when alive or even when dead? The first two questions were not included in the composite because they cannot be scored as correct or incorrect, given the lack of scientific consensus. The third question was not included in the composite because it was deemed redundant with the “dead-sick” question in the composite: “If a COVID-19 virus is dead, can it still make people sick?” Participants typically reported that viruses are alive (79% COVID-19, 80% Cold), they can die (81% COVID-19, 81% Cold), and they are dangerous only when alive (86% COVID-19, 82% Cold). For comparison, participants were highly accurate on the comparison questions regarding animals (horse, pig; 97% alive), plants (tree, grass; 89% alive), non-living natural kinds (cloud, moon; 86% not alive), moving artifacts (sled, bicycle; 95% not alive), and simple artifacts (hat, cup; 97% not alive). Altogether, these data suggest that participants construe viruses as biological entities. This is in striking contrast to the scientific dispute regarding the life status of viruses (given that viruses have only some qualities that are characteristic of living things). Relatedly, as noted earlier, a sizeable subset of participants incorrectly reported that viruses can grow bigger or move by themselves, suggesting that they may overextend certain biological features of animals to viruses.

### Coherence and Coexistence

To address the question of the coherence of people’s beliefs about viral transmission, including whether we see evidence of multiple explanatory frameworks, we focused on the qualitative coding of the responses to open-ended coding (see Table 4 for a summary of the data, including the mean number of explanations for which a code was used, as well as the percentage of participants who provided at least one response that received a given code). As can be seen, we again see consistent differences between the two conditions. Those in the COVID-19 condition compared to those in the Cold condition were significantly more likely to refer to germ movement (either explicitly or implicitly), less likely to endorse misconceptions (folk beliefs or undifferentiated illness), and more likely to explain vaccines as preventing disease. These results are consistent with the condition differences obtained in the Accuracy Composite, revealing higher levels of understanding for COVID-19 than the common cold. That participants invoked germ theory more for COVID-19 than Cold, and that folk theories were so much more frequent for Cold than COVID-19, are notable for again indicating that familiarity with a disease does not lessen the adherence to folk theories.

**TABLE 4:** Qualitative coding, mean # of responses receiving each code, with % of participants providing at least one code in brackets.

Concept	Code	COVID-19	Cold	COVID-19 vs. Cold
Transmission	Explicit germ movement	3.01 [82%]	2.06 [71%]	<0.001
	Implicit germ movement	1.96 [83%]	1.20 [71%]	<0.001
	Points of entry	0.66 [47%]	0.42 [37%]	n.s.
Biological processes	Virus survival or death	1.24 [65%]	1.13 [61%]	n.s.
	Virus replication	0.96 [62%]	1.12 [63%]	n.s.
	Viruses require a host	0.29 [21%]	0.44 [32%]	n.s.
	Immune system response	0.81 [51%]	1.08 [58%]	n.s.
Misconceptions	Folk beliefs	0.25 [19%]	1.07 [66%]	<0.001
	Undifferentiated illness	0.18 [15%]	0.59 [30%]	<0.001
Vaccines	Vaccines as preventive	0.65 [65%]	0.32 [32%]	<0.001
	Vaccines as curative	0.23 [22%]	0.12 [11%]	n.s.
Anthropomorphism	Anthropomorphism	1.46 [65%]	1.54 [73%]	n.s.

*Comparisons of COVID-19 vs. Cold conditions were conducted on the total number of responses, using paired-t tests (ps > 0.01 not reported, due to the multiple tests).*

#### Germ Theories and Folk Theories

Participants endorsed both germ theories and folk theories, often with both theories co-existing in the same individual. Overall, endorsement of germ theories was quite high, with roughly 82% of those in the COVID-19 condition and 71% of those in the Cold condition mentioning either implicit or explicit germ movement as responsible for illness. Nonetheless, participants also expressed inaccurate folk theories, most typically regarding weather or foods/vitamins as causative or curative. These beliefs were especially high in the Cold condition, expressed by nearly two-thirds of participants. Examples include: “they play in rain or drunk some cool drinks,” “not dressing properly,” or “climatic change” as causes of the common cold, and “drink hot water with pepper” or “take hot food and proper medicine” as curing the common cold. Although much rarer for COVID-19, folk beliefs were also expressed, for example, “I imagine many foods that bolster your immune system: garlic, green tea, ginger, etc. are great for prevention.” Some participants described folk beliefs and germ theory as alternative routes to getting sick, for example, that a person could get a cold “by contracting it from another person ***or*** getting cold too quickly after being hot” (emphasis added). Others incorporated folk beliefs into a scientific framework (e.g., “hot soups kill germs”; “Foods with vitamin C can strengthen your immune system to help you fight viruses”) or expressed folk beliefs alongside germ theory (e.g., “Keep your body filtered with high levels of vitamin C, stay away from sick people, wash hands frequently, remain out of cold weather without proper attire” as ways to prevent illness). Many others were mute on the relation between folk and scientific theories (e.g., “Peper [sic] is a best treatment for cold”; “vitamins” [help you get better from COVID-19]).

In order to assess co-existence of germ theory and folk theory, we examined how often individuals expressed both concepts in their open-ended responses. Specifically, we examined overlap between folk theories and three open-ended response codes that reflected endorsement of germ theory: explicit germ movement, germ replication, and immune system. We found that folk theories were endorsed by 40% of participants who explained illness in terms of the transmission of viruses (i.e., explicit reference to germ movement), 42% of those who spontaneously made reference to the immune system, and 37% of those who spontaneously mentioned viral replication. Conversely, of those expressing a folk theory, 71% made explicit reference to germ movement, 54% made reference to the immune system, and 54% made reference to viral replication. These patterns suggest that germ theory is predominant in this sample, but is not necessarily viewed as in conflict with folk theories.

Informal observation suggests a range of ways in which these seemingly competing beliefs may relate. Some participants endorsed different explanations depending on the question being asked (e.g., one participant said that people contract the common cold “through exposure to a chilly weather,” but when asked why social distancing is effective noted, “if I’m not close to such person, it reduces the chances of getting the germ”; another said that colds are caused by “eating sweet and … very cooling food, climate change, etc.” but when asked why hand-washing is effective said, “soap can kill virus bacterias [sic]”). Others juxtaposed different explanations in response to the same question (e.g., one gets a cold “From shifts in the weather and from being around others who have it”; “They get it when exposed to cold environments or being exposed to someone with a cold.”). Still others combined two explanations into a coherent whole (e.g., “I do think that some foods that are high in Vitamin C can help build your immune system to help your body fight the [COVID-19] infection if you do get it.”; “I imagine many foods that bolster your immune system: garlic, green tea, ginger, etc. are great for [COVID-19] prevention.”).

#### Anthropomorphic and Mechanical Explanatory Frameworks

We next examined how often participants made use of two different explanatory frameworks, namely ones that construed viral processes in either anthropomorphic or mechanical ways. Responses were coded as *anthropomorphic* if they referred to viruses or viral bodily processes (e.g., immune system response) as if they were agentive, intentional, psychological, or animate. Examples are provided in Table 5. The majority of participants used anthropomorphic language, often relying on anthropomorphism to talk about underlying (invisible) viral processes. These responses were especially common in response to questions regarding internal or invisible causal processes, including: why a virus is or isn’t alive (28% of participants), how a vaccine works (26% of participants), why there is a delay between exposure and symptoms (24% of participants), why a person feels sick all over their body (18% of participants), and why someone resting in a clean environment has germs in their body over time (13% of participants). In contrast, anthropomorphism was rarely used to talk about observable behaviors: how someone might contract a viral disease, how to protect against the disease, or why various behavioral practices (e.g., social distancing, washing hands) are effective (each between 0 and 2% of participants).

**TABLE 5:** Examples of anthropomorphic explanations (emphases added).

“Like all living organisms, it [virus] fights to survive.”
“The cold germ lives inside of your body, it then begins to replicate itself using your bodies [sic] cellular information. By the time it takes for this to happen your white blood cells have either not been informed or confused that the cold virus is safe to be in your body.”
“I suspect the germs died off from malnutrition in that time”
“The virus will be very happy with mucus membranes inside your mouth.”
“Soap is a disinfectant and the virus likes water droplets.”
“It is is cells trying to fight off the virus that causes inflammation.”

*Bolded terms indicate anthropomorphic wording.*

Although in some cases participants’ anthropomorphism may have reflected a confusion about the ontological status of a virus (participants sometimes referred to a virus as a single-celled organism, or reported that it could grow or eat), more typically it seemed to be a non-literal conceptual framework, for example, that the immune system “fights,” “battles,” or “attacks” the virus, or that a virus “likes” a certain environment. That is, participants seemed prone to use metaphor (Lakoff and Johnson, 1980) or analogy (Gentner et al., 2001) to provide a conceptual framework to explain the processes taking place in this domain. Participants’ use of psychological language or metaphors of battle or warfare entailed crossing ontological boundaries, such as referring to an immune system response (biological) while characterizing it as the body trying to fight off the virus (psychological and agentive). This appeared to be an effective strategy to explain how viruses operate. Indeed, participants who displayed greater knowledge about viral transmission were significantly *more* likely to anthropomorphize bodily processes, with rates of anthropomorphism correlating positively with scores on the accuracy composite, in both conditions (COVID-19: 0.34, *p* < 0.001; Cold: 0.28, *p* = 0.003). Anthropomorphism also correlated significantly with open-ended codes that primarily reflected greater understanding of the relevant biological processes, including germ survival (Cold: 0.26, *p* = 0.005), explicit movement (COVID-19: 0.31, *p* < 0.001; Cold: 0.33, *p* < 0.001), points of entry (COVID-19: 0.34, *p* < 0.001; Cold: 0.26, *p* = 0.004), germ replication (COVID-19: 0.32, *p* < 0.001; 0.35, *p* < 0.001), host (Cold: 0.25, *p* = 0.007), immune system (COVID-19: 0.39, *p* < 0.001; Cold: 0.48, *p* < 0.001), and vaccine as cure (COVID-19: 0.24, *p* = 0.009). None of the other codes correlated significantly with anthropomorphism.

Participants also at times appeared to use a *mechanical* explanatory framework rather than a biological one. Because we did not directly code for this type of response, the evidence here must be considered preliminary. Nonetheless, in our data, we noted that participants often expressed mechanical explanations when asked to explain why handwashing or cleaning a surface helps protect against COVID-19 or colds, by stating that these actions would remove germs by rinsing them off or wiping them away (rather than killing or destroying germs). Examples of such mechanical explanations included, “This [hand-washing] removes germs from your hands,” “It washes the virus off,” “This rids the germs off of hands so it won’t be spread to your mouth, eyes,” and “This will remove any germs that may be on the surface.” Overall, participants made reference to killing or destroying germs/viruses only 34% when asked to explain why washing hands is effective, 35% when asked to explain why cleaning a surface is effective, and 45% when asked to explain if washing hands without soap would protect one against disease.

Another set of items on which participants commonly appealed to a mechanical explanatory framework were those for which viral replication would be a relevant explanation: why there is a delay before symptoms emerge, why someone with COVID-19 or a cold would have more germs in their body after a few days, and why they would feel sick all over their body after a few days (see Table 1 for wording). (99% of participants’ explanations that were coded as mentioning viral replication were in response to these three questions.) Although the majority of participants indeed mentioned the biological process of viral replication in response to at least one of these three questions (62% of participants in the COVID-19 condition and 63% of participants in the Cold condition), when viral replication was not mentioned, participants often appealed instead to mechanical movement, mentioning that the virus can spread or travel (“It spread out to those parts”; “it takes time for the virus to circulate and infect a large enough portion of the person for them to feel unwell”; “They can go all over”; “They travel around the body in the bloodstream”).

### Attitudes, Behaviors, and Demographics

Of interest is also how participants’ attitudes, self-reported behaviors, and demographics link to their beliefs and explanations. Table 6 presents the mean scores for participants’ knowledge self-appraisal, desire for a vaccine, and (for COVID-19 condition only) attitudes, behaviors, and knowing someone who had been diagnosed with COVID-19. We pre-registered correlations among the following variables, separately for the COVID-19 and Cold conditions: accuracy (from the Accuracy Composite reported in the Accuracy section above), knowledge self-assessment, willingness to be vaccinated, and anthropomorphism. We also examined correlations with parent illness conversations and (for the COVID-19 condition only) COVID-19 attitudes and COVID-19 protective behaviors, as exploratory correlates. In accordance with our preregistration, we report only those correlations that are significant at *p* <0.01.

**TABLE 6:** Attitudes and behaviors, means (SDs) by condition.

	COVID-19	Cold
Knowledge self-appraisal		
OVERALL	3.24 (0.94)	3.03 (0.96)
Time 1	3.22 (1.02)	2.97 (1.10)
Time 2	3.25 (0.93)	3.10 (0.96)
Wanting a vaccine (scale range: 1–7)	5.23 (2.15)	4.31 (2.08)
Know someone diagnosed with COVID-19	31%	
COVID-19 attitudes (scale range: 1–4)		
OVERALL	3.53 (0.63)	
Social distancing effective	3.65 (0.68)	
Serious threat	3.61 (0.79)	
Confidence in medical scientists	3.34 (0.77)	
Protective behavior (scale range: 1-3)		
OVERALL	2.64 (0.43)	
Avoided going to public places	2.75 (0.57)	
Avoided small gatherings	2.76 (0.52)	
Avoided traveling by airplane	2.68 (0.69)	
Stocked up on food, medical, cleaning	2.33 (0.78)	
Wore mask when outside home	2.76 (0.57)	
Maintained 6 feet of distance	2.82 (0.48)	
Practiced frequent hand-washing	2.77 (0.53)	
Avoided touching face	2.65 (0.63)	
Avoided contact with sick	2.79 (0.50)	
Covered coughs, sneezes	2.80 (0.51)	
Cleaned/disinfected surfaces	2.69 (0.59)	
Cleaned/wiped down groceries	2.45 (0.78)	
Worked from home	2.55 (0.74)	
Stayed home from work	2.18 (0.91)	

#### Knowledge Self-Appraisal

Although participants were more accurate on the COVID-19 survey than the Cold survey, the Self-knowledge scale did not differ between conditions, *M*s = 3.24 (0.94) and 3.03 (0.96) for COVID-19 vs. Cold respectively, *t*(236) = 1.64, *p* = 0.103. We were also interested in whether going through the in-depth survey would lower participants’ confidence in their knowledge, given the illusion of explanatory depth (Rozenblit and Keil, 2002), but this was not the case. Those in the Cold condition actually rated their knowledge as higher after taking the survey (time 1 *M* = 2.97 [1.10]; time 2 *M* = 3.10 [0.96]), *t*(118) = –2.03, *p* = 0.045, whereas those in the COVID-19 condition showed no difference (time 1 *M* = 3.22 [1.02]; time 2 *M* = 3.25 [0.93], *t*(118) = –0.67, *p* > 0.5).

Participants’ knowledge self-appraisal correlated negatively with their accuracy scores (on the Accuracy Composite), in both conditions (COVID-19: –0.29, *p* = 0.001; Cold: –0.40, *p* < 0.001). In other words, those who knew more seemed to be more aware of their knowledge gaps than those who knew less. This is consistent with the Dunning-Kruger effect, in which people are often unaware of what they don’t know (Dunning, 2011).

#### Wanting a Vaccine

Participants in the COVID-19 condition reported wanting a COVID-19 vaccine more than those in the Cold condition reported wanting a Cold vaccine, *M*s = 5.23 (2.15) and 4.31 (2.08), respectively, *t*(236) = 3.34, *p* < 0.001. For COVID-19, 68% of participants indicated that they would want a COVID-19 vaccine (scoring 5, 6, or 7 on the 7-point scale), 23% indicated that they would not (scoring 1, 2, or 3), and 9% were unsure (scoring 4). For the common cold, 47% indicated that they would want a cold vaccine, 30% indicated that they would not, and 23% were unsure. Those in the Cold condition often explained not wanting a vaccine by mentioning that a cold is too minor to warrant a vaccine, or that there are too many cold variants for a vaccine to be useful. For the COVID-19 condition, but not the Cold condition, vaccine willingness correlated positively with Accuracy (i.e., score on the Accuracy Composite), Pearson’s *r* = 0.32, *p* < 0.001.

#### Knowing Someone Diagnosed With COVID-19

Participants in the COVID-19 condition who reported personally knowing someone who had been officially diagnosed with COVID-19 provided higher knowledge self-appraisal scores than those who reported not knowing such a person [*M*s = 3.50 (0.79) vs. 3.12 (0.98), *t*(117) = 2.10, *p* = 0.038], but were lower on the accuracy composite [*M*s = 0.77 (0.21) vs. 0.87 (0.12), *t*(117) = –3.08, *p* = 0.003]. There were no differences between the two groups in their COVID-19 attitudes, protective behaviors, or interest in being vaccinated.

#### Parent Illness Conversations

We next examined the frequency of self-reported conversations that parents had with their children. We were interested in how often parents reported discussing different aspects of viral illness, and whether this varied as a function of illness type. We therefore conducted a 3 (conversational topic: definition, prevention, consequences) × 2 (condition: COVID-19, Cold) ANOVA. This yielded a main effect of Condition, *F*(1,111) = 3.99, *p* = 0.048, η_*p*_^2^ = 0.035, a main effect of topic, *F*(2,222) = 11.16, *p* < 0.001, η_*p*_^2^ = 0.09, and a condition × topic interaction, *F*(2,222) = 7.12, *p* = 0.001, η_*p*_^2^ = 0.06. Pairwise comparisons of the interaction revealed that parents were equally likely to have conversations about definitions for COVID-19 and the common cold [*M*s = 2.93 (0.14) and 2.93 (0.13), respectively], *p* > 0.9, but that they were more likely to discuss prevention and consequences for the common cold [*M*s (*SD*s) = 3.55 (0.14) and 3.26 (0.14), respectively] than for COVID-19 [*M*s (*SD*s) = 3.02 (0.14) and 2.75 (0.15), respectively], *p*s < 0.02. Finally, parents who reported knowing someone diagnosed with COVID-19 were more likely to report engaging in conversations with their children about illness [*M*s = 3.37 (1.15) vs. 2.68 (1.10), *t*(53) = 2.11, *p* = 0.04].

#### Attitudes and Protective Behaviors

As noted above, attitudes and protective behaviors were assessed only for those in the COVID-19 condition, and thus all results involving attitudes and protective behaviors are for that condition only. We found that COVID-19 attitudes (e.g., viewing COVID-19 as a serious threat; trust in medical scientists) correlated positively with scores on the accuracy composite (0.25, *p* = 0.006), with explaining vaccines as preventing disease (open-ended response; 0.33, *p* < 0.001), and with interest in being vaccinated (0.47, *p* < 0.001). Engaging in protective behaviors against COVID-19 correlated negatively with folk beliefs (open-ended response; –0.28, *p* = 0.002), and positively with the accuracy composite (0.44, *p* < 0.001), with mention of germ survival (open-ended response; 0.28, *p* = 0.001), and with interest in being vaccinated (0.34, *p* < 0.001). Finally, COVID-19 attitudes and protective behaviors correlated highly with one another, indicating that those who took COVID-19 seriously were more likely to report engaging in protective behaviors (0.60, *p* < 0.001).

#### Demographics

As pre-registered, we examined how accuracy related to demographic variables. We did not find any significant correlation with education, income, age, or voting in respondent’s zip code, for either the COVID-19 or Cold condition. Accuracy also did not differ by gender, for either condition.

## Discussion

This exploratory study provided an in-depth examination of adults’ causal reasoning about two viral diseases, COVID-19 and the common cold, during the first year of the COVID-19 pandemic. Our primary focus is what these findings might reveal about when, why, and how folk theories and scientific theories relate to one another, in these two particular test cases. It is important to be mindful that there are many other examples of folk/scientific theory enmeshment beyond these two diseases, and in other content domains. Key themes that emerged include that, for many participants, viral and non-viral explanatory beliefs co-exist within their explanations for viral illness; multiple causal frameworks are simultaneously employed (which may variously be considered ontologies, explanatory stances, or modes of construal); and causal understandings correlate with participants’ attitudes and behaviors. We also noted that there are limits to participants’ metacognitive knowledge (what they know about what they know), which may have implications for the messages they pass along to others. Below we discuss each of these points in turn, followed by conclusions that consider the implications for formal models of explanatory reasoning, and questions for the future.

### Co-existence of Viral and Non-viral Explanatory Beliefs

The present findings indicate that folk theories do not disappear with greater familiarity or experience, and if anything, folk theories may increase as there are more opportunities for generating, communicating, and transmitting alternative accounts. The majority of participants endorsed a germ theory of viral illness and were highly accurate on forced-choice questions regarding the viral transmission process. They indicated knowledge of non-obvious aspects of viral disease, including that carriers can be asymptomatic, that seemingly innocuous behaviors like singing with others can transmit disease, that there is a delay between exposure to the virus and showing symptoms, that reinfection is possible, and that a virus can enter the body through the eyes. At some point during the survey, most participants made either explicit or implicit reference to germ movement or germ transfer to explain viral transmission.

Nonetheless, alongside this understanding of viral transmission, many participants also expressed non-viral folk theories, such as cold weather or particular foods as causing or curing illness. For example, non-viral folk theories were endorsed by roughly 40% of participants who explained illness in terms of the transmission of viruses, made reference to the immune system, or mentioned viral replication. How people combined these seemingly competing beliefs varied (see also Legare and Gelman, 2008; Legare et al., 2012), at times simply juxtaposing different explanations, at times using different explanations for different phenomena, and at times integrating folk theories into scientific theories (e.g., reporting that exposure to cold would weaken a person’s immune system; see also Au et al., 2008).

Although comparisons of COVID-19 and the common cold revealed many commonalities, the latter, more familiar disease elicited consistently lower levels of accuracy, less mention of germ transmission, and greater mention of folk theories. The greater reliance on folk theories for the more familiar disease of the common cold is consistent with some studies that find that adults are more likely to endorse non-scientific theories than children (Raman and Winer, 2004; Legare and Gelman, 2008). It may also be that folk theories of COVID-19 took time to emerge, and were relatively less available in the summer of 2020, when this study was conducted. However, it may also be that the present study underestimated folk theories for COVID-19 because the survey did not ask about any of the more prevalent theories that have been put forward (see https://www.hsph.harvard.edu/india-center/myths-vs-facts/ for a host of examples).

An important question is why we obtained different patterns of results for the two diseases. We had assumed that COVID-19 is anomalous relative to the common cold, given its novelty, danger, and variability (as sketched out in the introduction). One possibility going into this study was that the anomalous nature of COVID-19 would prompt a proliferation of folk theories to step in where there were gaps in the scientific evidence. The data would argue against this possibility. However, another possibility is that the anomalous nature of COVID-19 made people more receptive to scientific evidence, at least initially (i.e., during the initial months of the pandemic, when this study was conducted). Alternatively, it may be that the anomalous nature of COVID-19 played little role, and that overall better performance in the COVID-19 condition was due to the tremendous amount of media coverage regarding COVID-19 at the time of data collection. These questions remain for future research.

### Multiple Causal Frameworks

The causal frameworks used to understand a complex domain can have consequences for people’s inferences and decision-making. For example, whereas a biological causal framework of contagious disease draws attention to conditions that foster germ reproduction versus death and thus effective means of destroying germs (e.g., washing utensils in hot, soapy water), a mechanical causal framework draws attention to the movement of germs in space, leading to ineffective behaviors (e.g., trying to “rub off” germs with a napkin; Au et al., 2008). Thus, to the extent that participants maintain multiple causal frameworks, this may stand in the way of appropriate inferences and decisions about how to prevent disease spread.

In the present study, in addition to expressing both viral and non-viral explanations for disease, participants made use of multiple explanatory frameworks (biological, mechanical, and psychological) when explaining how viruses operate. A biological framework predominated, with nearly all participants judging that viruses are alive (in contrast to scientists, who characterize viruses as quasi-living agents; Villarreal, 2004), and in their open-ended responses making reference to biological processes or entities that included germ survival or death, germ replication, and the body’s immune system. This is in contrast to young children, who have been found to construe germs as equivalent to poisons rather than as biological entities (Solomon and Cassimatis, 1999). Interestingly, some participants in the present study overextended a biological framework, referring to viruses as needing food, or having the ability to grow bigger in size or move by themselves.

We also found evidence that many participants made use of mechanical frameworks to explain biological processes (e.g., explaining the effectiveness of hand-washing or cleaning counters as “wiping off” germs rather than destroying them, or explaining symptom delay as due to germs traveling through the body rather than replicating). These findings are consistent with extensive research conducted by Au and Romo (1999) and Au et al. (2008), who found that both children and older adults in China and the U.S. tended to explain viral illness in terms of mechanical causal frameworks (how germs move or are moved around) rather biological ones (germ survival and reproduction). It would be valuable in future work to examine the extent to which mechanical explanations represent a less advanced theory that is replaced by biological explanations, versus the extent to which both explanatory systems co-exist.

Additionally, we were interested in when and how participants made use of a non-literal, anthropomorphic framework (attributing psychological, intentional, or agentive attributes) as a means of framing viral disease processes (e.g., describing the immune system as trying to fight off a virus). The role of anthropomorphic language in scientific reasoning is a source of debate. On the one hand, anthropomorphism has been viewed negatively, as leading to inaccurate concepts and inferences even among scientists (Martin, 1991; Davies, 2010). On the other hand, anthropomorphic language has been argued to make complex scientific concepts more familiar, approachable, and memorable, and thus resulting in more sustainable learning (Zohar and Ginossar, 1998; Kattmann, 2008; Stoos and Haftel, 2017). In our data, a striking finding was that anthropomorphic language was used not in contrast to biological explanations, but rather to express them. Indeed, roughly two-thirds of the participants used such language at least once, and participants who used anthropomorphic language displayed significantly *greater* knowledge about viral transmission in their responses to the close-ended questions in the accuracy composite. Because a challenge in understanding viral disease is that the structures, processes, and mechanisms are not directly visible, Jee et al. (2015) suggest that analogies, such as “An infection is like a war,” may scaffold people’s understanding.

In accord with Keil (2006), we suggest that anthropomorphism may be thought of as a “stance” or “mode of construal” (as when someone describes magnets as “liking” certain metals, or substances as have “memories”). Keil suggests that such construal are too vague and non-predictive to be considered full-fledged theories, although this does not mean that they have no conceptual consequences. He notes that framing a phenomenon from different stances or modes of construal can yield both insights and distortions (see also Jee et al., 2015). In the case of viral illness, for example, consider participants who talked about the virus “finding” a host or hospitable environment (“It took a few days for the virus to set up shop and find a friendly environment to grow in and to a size that affected the man”; “Washing your hands removes the virus from them so they can not find a way inside”; “It takes a while for the virus to find and attach to a cell and take it over”). This framing the virus as active and on the lookout for a home may contribute to thinking about a virus as goal-directed and agentive (see also Osbeck and Nersessian, 2011, 2013).

### Attitudes and Behaviors

A number of scholars have suggested that causal theories can be influenced by factors such as attitudes and trust in authority. For example, Chinn and Brewer (1993) suggest that people may endorse beliefs that satisfy strong personal or social goals. Similarly, Keil (2006) notes that one way to deal with gaps in explanatory understanding is to outsource the explanation to someone else who is more knowledgeable—a step that requires deciding on who to trust. In our data, we were able to examine this question within the COVID-19 condition only, as only those participants received questions regarding attitudes and protective behaviors. We found that accuracy positively correlated with attitudes (trusting medical scientists and taking the disease more seriously), self-protective behaviors (such as social distancing and mask-wearing), and willingness to be vaccinated against COVID-19 (note that this was asked before a vaccine had been developed). This is consistent with Thoma et al. (2021), who found that knowledge about disease mechanism for COVID-19 correlated with degree of self-reported precautionary behaviors, such as hand-washing and wearing face masks. (Interestingly, they found that simply knowing COVID-19 symptoms did not correlate with precautionary behaviors.) It is also consistent with Motoki et al. (2021), who found that scientific literacy was linked to attitudes toward COVID-19 vaccinations, and Sanchez and Dunning (2021), who found that attitudes regarding scientists predicted COVID-19 attitudes and protective behaviors even more than did political partisanship.

The direction of influence in our data remains unclear. One possibility is that those who took COVID-19 more seriously not only engaged in more self-protective behavior but also were more motivated to learn more, thus seeking out more information and leading to higher accuracy scores. Another possibility is that those who were better informed to begin with were more aware of the risks of COVID-19 and thus more motivated to engage in self-protective behavior. It is also quite possible that the links we obtained were due to some third variable, such as political attitudes (see Pennycook et al., 2021). We did not find significant correlations with demographic variables in our own data, though that may be because we did not include a measure of participants’ political views.

### Limits of Metacognitive Knowledge

In addition to assessing participants’ knowledge, we twice asked participants to indicate how knowledgeable they were about how the COVID-19 virus works [COVID-19 condition], or how cold viruses work [Cold condition]: once at the beginning of the survey, and once at the end. In both conditions, we found a seemingly counter-intuitive effect that accuracy negatively correlated with self-assessed knowledge – in other words, those who knew more were less confident in their knowledge. This could reflect the Dunning-Kruger effect (Dunning, 2011), whereby people with limited knowledge or ability overestimate their knowledge or competence. As Dunning (2011) notes, “many instances of ignorance may be obscured because they are hidden behind misbeliefs that people mistake for valid knowledge in the domain in question.” It is also possible that those who know more are appropriately aware that there is much that is still unknown about viruses. An important question for future research is the nature of information that people communicate to others, both adults in one’s social networks (actual and virtual) and children who seek information and reassurance from their parents (see Menendez et al., 2021, for an in-depth examination of the latter). Given that those who are confident in their beliefs are more likely to hold misleading or inaccurate theories, misinformation may be projected with confidence and certainty that encourages others to trust and amplify such beliefs.

An additional potential contributor to limited metacognitive awareness may be an illusion of explanatory depth (Rozenblit and Keil, 2002), whereby people erroneously believe they understand how a system or device works (e.g., how a helicopter flies), but in reality have only a crude or superficial sketch (e.g., it has something to do with the blades rotating). Consistent with this possibility, we noted that some participants provided vague explanations that were lacking in mechanistic detail (e.g., stating that “medicine” or “pills” could help treat COVID-19 or the cold; noting that the immune system would fight the virus but not explaining how; or stating that a vaccine “protects people from getting attacked by germs”). These may be considered “placeholder” explanations.

With this in mind, we had predicted that participants would rate their knowledge as higher at the beginning of the survey than at the end, because prior research indicated that when people are confronted with the limits of their knowledge, this reduces the illusion. In contrast, we did not find any decline in people’s knowledge self-assessment after responding to the survey questions, though it may be that responding to the survey questions did not sufficiently indicate their lack of knowledge. In order for people to realize the limits of their understanding, it may be necessary to challenge their explanations more directly.

## Conclusion

In the current study, participants evidenced high levels of accuracy regarding the basics of the viral transmission process—although, unexpectedly, these patterns were less evident in people’s reasoning about the more familiar disease (the common cold) than the novel disease that had emerged as a pandemic only 3–4 months prior to the study was conducted (COVID-19). It is unclear whether the relative advantage of COVID-19 reflects a more general pattern regarding disease familiarity, or something special about these two particular illnesses. Future research could more systematically investigate diseases varying in familiarity. An additional interesting question for the future is whether accuracy in reasoning about COVID-19 has increased over time (given more experience with the disease) or decreased over time (given the opportunity for culturally specific alternative explanatory models to emerge; Legare et al., 2012).

The data also revealed complexities in people’s reasoning, consistent with individuals appealing to multiple explanatory frameworks, including the co-existence of viral and non-viral explanatory accounts; multiple stances or modes of construal wherein viruses were variously characterized as biological, psychological, and/or mechanical entities; and at times a reliance on placeholder concepts that gestured toward an explanation without providing a mechanistic account. The presence of multiple frameworks supports a growing body of evidence that lay understandings of science may be fragmentary, conflicting, and/or inconsistent (see also Chinn and Brewer, 1993; Di Sessa et al., 2004; Keil, 2006; Koslowski, 2013; Shtulman and Legare, 2020; Murray et al., 2021, for fuller discussion). As a consequence, this may foster disengagement with public health recommendations, due to a lack of understanding of how changes in behaviors can result in more positive outcomes (Prochaska, 2020), as for example when people hold erroneous beliefs regarding vaccine safety and/or efficacy. Moreover, even when people do wish to follow expert advice in order to avoid disease transmission, misconceptions may stand in the way of effective decision-making (as, for example, when people rely on folk theories of illness, or construe viruses in terms of mechanical rather than biological processes; Au et al., 2008). Nonetheless, it is important to consider that, as Weisman and Markman (2017) have suggested, people do not need access to fully detailed (or, we might add, fully coherent) causal accounts in order for them to appreciate the reasons underlying the effectiveness of health-promoting behaviors.

We also found that attitudes and motivated beliefs appeared to play a role in people’s predictions and explanations regarding COVID-19, consistent with other recent reports in the literature (e.g., Motoki et al., 2021; Sanchez and Dunning, 2021; Thoma et al., 2021). In the current data, trust in medical science and taking the COVID-19 disease seriously were predictive of people’s accuracy in explaining the disease, as well as their understanding of vaccines and with wanting to be vaccinated. Engaging in self-protective behaviors against COVID-19, such as wearing masks and social distancing, also correlated with accuracy and with wanting to be vaccinated, as well as negatively predicting use of non-viral folk theories. Why these correlations obtained is not yet clear. Nonetheless, the findings provide further support for the suggestion that interventions to encourage greater adherence to public health recommendations likely will need to target a range of factors that contribute to distrust of scientific evidence (e.g., Chinn et al., 2021; Kubin et al., 2021; Murray et al., 2021), in addition to misconceptions and informational gaps in lay theories (Weisman and Markman, 2017).

## Data Availability Statement

The raw data supporting the conclusions of this article will be made available by the authors, without undue reservation.

## Ethics Statement

The studies involving human participants were reviewed and approved by University of Michigan Health Sciences and Behavioral Sciences Institutional Review Board (IRB-HSBS). The patients/participants provided their written informed consent to participate in this study.

## Author Contributions

DL and SG contributed to the conception and design of the study and performed the data analysis. DL performed the research and organized the data. SG wrote the first draft of the manuscript. Both authors contributed to the manuscript revision, read, and approved the submitted version.

## Author Disclaimer

Any opinions, findings, and conclusions or recommendations expressed in this material are those of the authors and do not necessarily reflect the views of the National Science Foundation.

## Conflict of Interest

The authors declare that the research was conducted in the absence of any commercial or financial relationships that could be construed as a potential conflict of interest.

## Publisher’s Note

All claims expressed in this article are solely those of the authors and do not necessarily represent those of their affiliated organizations, or those of the publisher, the editors and the reviewers. Any product that may be evaluated in this article, or claim that may be made by its manufacturer, is not guaranteed or endorsed by the publisher.
